# Identification of Histopathological Biomarkers in Fatal Cases of Coronavirus Disease: A Study on Lung Tissue

**DOI:** 10.3390/diagnostics13122039

**Published:** 2023-06-12

**Authors:** Ioana-Andreea Gheban-Roșca, Bogdan-Alexandru Gheban, Bogdan Pop, Daniela-Cristina Mironescu, Vasile Costel Siserman, Elena Mihaela Jianu, Tudor Drugan, Sorana D. Bolboacă

**Affiliations:** 1 Department of Medical Informatics and Biostatistics, Iuliu Hațieganu University of Medicine and Pharmacy, 400349 Cluj-Napoca, Romania; andreea.gheban-rosca@umfcluj.ro (I.-A.G.-R.); tdrugan@umfcluj.ro (T.D.); 2 Clinical Hospital of Infectious Diseases, 400003 Cluj-Napoca, Romania; 3 Rouen University Hospital—Charles-Nicolle, 76000 Rouen, France; 4 Department of Histology, Iuliu Hațieganu University of Medicine and Pharmacy, 400347 Cluj-Napoca, Romania; marina.elena@umfcluj.ro; 5 The Oncology Institute “Prof. Dr. Ion Chiricuță”, 400015 Cluj-Napoca, Romania; bogdan.pop@umfcluj.ro; 6 Department of Anatomic Pathology, Iuliu Hațieganu University of Medicine and Pharmacy, 400349 Cluj-Napoca, Romania; 7 Institute of Legal Medicine, 400006 Cluj-Napoca, Romaniacsiserman@umfcluj.ro (V.C.S.); 8 Department of Forensic Medicine, Iuliu Hațieganu University of Medicine and Pharmacy, 400006 Cluj-Napoca, Romania

**Keywords:** histopathology biomarker, severe acute respiratory syndrome coronavirus 2 (SARS-CoV-2), coronavirus disease (COVID-19), autopsy, lung

## Abstract

We aimed to evaluate the primary lung postmortem macro- and microscopic biomarkers and factors associated with diffuse alveolar damage in patients with fatal coronavirus (COVID-19). We retrospectively analyzed lung tissue collected from autopsies performed in Cluj-Napoca, Romania, between April 2020 and April 2021 on patients with severe acute respiratory syndrome coronavirus 2 (SARS-CoV-2). We examined 79 patients with confirmed SARS-CoV-2 infection, ages 34 to 96 years, split into two groups using the cut-off value of 70 years. Arterial hypertension (38%) and type 2 diabetes mellitus (19%) were the most common comorbidities with similar distribution between groups (*p*-values > 0.14). Macroscopically, bloody exudate was more frequently observed among patients < 70 years (33/36 vs. 29/43, *p*-value = 0.0091). Diffuse alveolar damage (53.1%) was similarly observed among the evaluated groups (*p*-value = 0.1354). Histopathological biomarkers of alveolar edema in 83.5% of patients, interstitial pneumonia in 74.7%, and microthrombi in 39.2% of cases were most frequently observed. Half of the evaluated lungs had an Ashcroft score of up to 2 and an alveolar air capacity of up to 12.5%. Bronchopneumonia (11/43 vs. 3/36, *p*-value = 0.0456) and interstitial edema (9/43 vs. 2/36, *p*-value = 0.0493) were significantly more frequent in older patients. Age (median: 67.5 vs. 77 years, *p*-value = 0.023) and infection with the beta variant of the virus (*p*-value = 0.0071) proved to be significant factors associated with diffuse alveolar damage.

## 1. Introduction

Severe acute respiratory coronavirus 2 (SARS-CoV-2) is known to produce coronavirus-19 (COVID-19), a pathology classified as a multisystemic disease [1] that primarily affects the lungs [2,3]. Upon entering the host organism, SARS-CoV-2 binds to the angiotensin-converting enzyme-2 (ACE2) receptor on the cell surface, allowing the virus to enter the cell and promote replication. The ACE2 receptors are expressed in several tissues, including the heart, lungs, and kidneys [4,5].

In mild cases of infection, SARS-CoV-2 primarily causes lower respiratory tract infections (LRTIs) and severe pneumonia. In advanced cases, the disease can lead to acute respiratory distress syndrome (ARDS), septic shock, multiple organ dysfunction syndrome (MODS), and eventually death [6]. Due to the absence of adequate biosafety measures during the initial stages of the pandemic, there were limited histopathological studies conducted, which prevented the medical community from understanding the full extent of the virus’s effect on humans [7]. The number of postmortem examination studies is still limited, even though they are an invaluable tool for determining the pathogenesis of any disease, including COVID-19. Furthermore, autopsies can provide additional data to improve clinical care and treatment strategies [8]. Although the virus can cause lesions to other organs and tissues, lung changes are the most severe and will be this study’s primary area of focus.

The lung lesions caused by the SARS-CoV-2 virus previously reported in the scientific literature are summarized and briefly presented in Table 1. The table provides a clear overview of the macroscopic and microscopic changes that occur in the lungs due to infection, making it easier to understand the impact of the virus on this vital organ. Despite widespread vaccination campaigns and public health interventions in many countries, the virus continues to spread, leading to illnesses, hospitalizations, and fatalities. New virus variants are emerging, some more transmissible and potentially more resistant to vaccines, making it harder to control the ongoing pandemic [9].

Histopathological findings can accelerate the identification of effective epidemiological and medical strategies to reduce the progression of a pandemic caused by a novel virus with rapid and exponential transmission rates. A higher fatality rate has been reported in older subjects, but histological differences among different age groups were insufficiently documented. Furthermore, limited evidence exists on factors associated with microscopic diffuse alveolar damage. Our study had a two-fold aim: first, to identify the primary lung postmortem macroscopic and microscopic biomarkers observed in patients who died of COVID-19 using standard, special staining, and digital microscopy techniques in relation to age, and second,, to identify and evaluate if there are any factors associated with microscopic diffuse alveolar damage.

## 2. Materials and Methods

The study followed the Declaration of Helsinki, and its protocol was approved by the Ethics Committee of the Iuliu Hațieganu University of Medicine and Pharmacy Cluj-Napoca (DEP67/14.12.2021) and of the Institute of Legal Medicine, Cluj-Napoca (2406/XII/703/24.03.2022).

### 2.1. Study Settings and Design

We conducted an observational cohort study. All autopsies performed by forensic pathologists at the Institute of Legal Medicine Cluj-Napoca, Romania, were evaluated on patients who tested positive for SARS-CoV-2 ante- or postmortem between April 2020 and April 2021.

We included in our study only patients with COVID-19 confirmed disease regardless of the comorbidities, to whom an autopsy was performed in our institute in the study time-frame, and the lung tissue blocks were available from both lungs. We excluded patients with uncertain COVID-19 diagnosis from the analysis and those with confirmed COVID-19 disease with autopsy but without lung tissue blocks harvested from both lungs.

Demographic (sex, age, and living environment), epidemiological (symptoms onset, symptomatology duration, and date of death), and clinical (overlapping infections, mechanical ventilation) data were collected retrospectively from forensic reports. Comorbidities (class of body mass index, chronic obstructive pulmonary disease, arterial hypertension, congestive heart failure, myocardial infarction, diabetes mellitus, autoimmune diseases, liver steatosis, hepatitis, cirrhosis, chronic renal disease, malignant tumors, and brain stroke) were documented based on the available clinical data and the morphological observations made during autopsies.

### 2.2. Histological Processing and Staining

The harvested lung tissues were fixed in 10% neutral buffered formalin, processed, and paraffin embedded. The paraffin block was sectioned at 0.5 μm. Slides were manually stained using hematoxylin and eosin to highlight the cells, nuclei, and trichrome Masson to visualize collagen fibers and evaluate interstitial fibrosis.

Trichrome Masson’s special staining implied deparaffinization and rehydration through successive baths of 100%, 95%, and 70% alcohol. The lung tissue was rinsed and then stained in Wiegert’s iron hematoxylin for 10 min, rinsed, washed, and stained in Biebrich scarlet-acid fuchsin solution, and washed and differentiated in the phosphomolybdic-phosphotungstic acid solution until the collagen changed color. The lung tissue was dehydrated, cleared in xylene, and mounted on a microscopic slide.

### 2.3. Microscopic Examination

One independent board-certified pathologist evaluated the lung tissue samples and was blinded to patient characteristics and diagnoses. A Leica DM2500 microscope (Buffalo Grove, IL, USA) was used to examine the H&E slides. The findings were evaluated based on standard histopathological practice [18,19,20].

The trichrome Masson stained slides were examined as digital whole slide images. The extent of fibrosis in the lung parenchyma was evaluated using the Ashcroft score [21], a semi-quantitative scoring system that assesses the degree of lung fibrosis on histopathological digital slides. The Ashcroft score ranges from 0 to 8, based on the degree of fibrous thickening of the alveolar walls, lung architecture damage, and small fibrous proliferative bundles [21]. Moreover, the number of fibroblastic bundles was manually counted in a 25 cm^2^ area of lung tissue.

### 2.4. Morphometric Analysis

**Figure 1:**
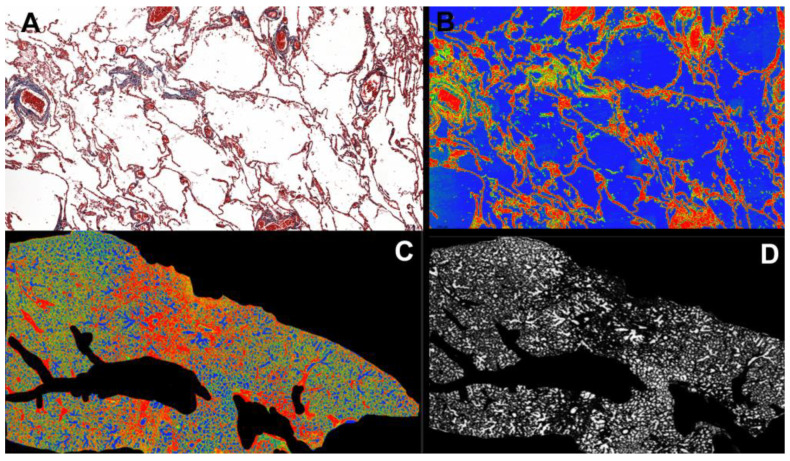
(**A**). Pulmonary parenchyma in Trichrome Masson stain at 20× magnification. (**B**). Red Green Blue (RGB) conversion using the Gradient Map Visualisation tool (SlideViewer by 3DHISTECH) highlights alveolar air space in the blue spectrum. (**C**). Overall view of the pulmonary fragment at 0.5× magnification, in RGB, after cropping vascular spaces using Adobe Photoshop CS9. (**D**). Pulmonary fragment after RGB stacking using ImageJ/Fiji software (version 2.13.1), highlighting only the free alveolar spaces in a white gradient to measure the total air-filled alveolar percentage of entire lung fragment tissue.

Whole Slide Images were obtained by scanning the physical trichrome Masson’s slides using a Pannoramic SCAN 150 by 3DHISTECH (Budapest, Hungary). The average scanning parameters were: 20× magnification, using a Plan-Apochromat objective and a CIS VCC camera with a micrometer/pixel ratio of 0.194475, scan duration of 17 min, calibrated color scheme, 112,640 × 243,200-pixel slide dimension, 9454 scanned fields of view, file size 2.72 GB. Measurement of the percentage of alveolar space was performed using the software SlideViewer by 3DHISTECH (version 2.6, Budapest, Hungary) (SlideViewer, RRID: SCR_017654). Using the plugin “Gradient Map Visualizations” we converted the trichrome Masson microscopic slides into an RGB spectrum (RedGreenBlue) slides where red and green highlighted interstitial and vascular structures and blue highlighted the alveolar space. We took snapshots of the pulmonary section slides and saved them as TIFF.files. Adobe Photoshop CC 2019 (San Jose, CA, USA) (Adobe Photoshop, RRID: SCR_014199) was used to crop and edit the image only to include the lung parenchyma without vascular lumina, which had the same color as alveolar spaces. The digital analysis was performed using the image analysis software ImageJ/Fiji (version 2.13.1, LOCI, University of Wisconsin, Madison, WI, USA) (ImageJ, RRID: SCR_003070) with the RGB Stack option to make a montage and adjust the color threshold of BLUE to 100–250 and analyzed the image by the following measurements: area, area fraction, and limit threshold. The same optical and image parameters, scan settings, and hardware versions were used to evaluate all images. The user defined the area of interest (ROI), and the software retrieved the percentage of free alveolar space from the examined section (Figure 1).

### 2.5. Statistical Analysis

We classified patients as infected with variant alpha (B.1.1.7) or beta (B.1.351, starting from March 2021) according to information available in Global Initiative on Sharing All Influenza Data (GISAID) [22].

We divided the cohort according to age into two groups (<70 years vs. ≥70 years), and we compared demographic, macroscopic, and microscopic characteristics between the two groups.

Shapiro–Wilk test (*p* < 0.05) and Q-Q plots were used to assess the normality of quantitative variables. Continuous data exhibiting non-normal distribution were described as the median and interquartile range (IQR). Categorical variables are summarized as absolute frequencies and percentages. Fisher or Chi-squared tests were applied to test associations in contingency tables based on the appropriate counts of the expected frequency tables. Differences between investigated groups were evaluated with non-parametric tests (Mann–Whitney U test) for continuous variables with non-normal distribution. All tests were two-sided, and the results were considered significant at *p*-values < 0.05.

Logistic regression was applied to identify factors related to diffuse alveolar damage (DAD) occurrence. Those factors who were associated with DAD at a *p*-value less than 0.25 in uni-variable models [23] were included in the multivariable regression model using stepwise selection, forward Wald.

Statistical description and analyses were conducted using Simple Interactive Statistical Analysis (SISA by Quantitative Skills, Available Online: https://www.quantitativeskills.com/sisa/ (accessed on 7 March 2023)), IBM SPSS trial version (Armonk, NY, USA) (IBM SPSS Statistics, RRID: SCR_019096), and Microsoft Office Excel 365 (Redmond, WA, USA) (Microsoft Excel, RRID: SCR_016137).

## 3. Results

### 3.1. Patients Characteristics

Seventy-nine patients aged between 34 and 96 years old, 36 (45.6%) younger than 70, and 43 (54.4%) older than 70, were evaluated. Among the 79 patients included in the analysis, 51 (64.6%) died in 2020, and 28 (35.4%) died in 2021. Men (median age of 70, IQR = [60 to 79]) were statistically significant younger (Mann–Whitney test: Z statistics = −2.2, *p*-value = 0.0263) than women (median age of 80, IQR = [68 to 85]). Six patients (7.6%) were under the age of 50, ten patients (12.7%) were between the ages of 50 to 59, and twenty patients (25.3%) were 60 to 69 years old. Most deaths, 51.9% (n = 41), occurred in the hospital setting, followed by 25.3% (n = 20) at home and 13.9% (n = 11) in an ambulance. The place of death could not be determined for one patient. Data regarding days from symptom onset to death was available only for 25 cases and ranged from 1 day to 35 days.

The two investigated groups were similar regarding demographic characteristics, as shown in Table 2.

The number of comorbidities varied from none to nine, without a significant association between the presence of comorbidities (any) and sex (χ^2^ = 0.1, *p*-value = 0.7509). The top three most frequent comorbidities observed in the cohort were: arterial hypertension (38%), type 2 diabetes mellitus (19%), and congestive heart failure (12.7%). A clinical history of myocardial infarction was observed exclusively in patients older than 70. Only one man, 72 years old, had a clinical diagnosis of liver cirrhosis; another man, 46 years old, had chronic renal disease. Fifteen (19%) patients had mechanical ventilation, eleven (25.6%) being in the group older than 70, but the difference did not reach the statistical significance threshold (χ^2^ = 2.7, *p*-value = 0.1024).

Regarding the microbiological results, two patients had positive sputum cultures for *Acinetobacter baumannii*, and another showed the presence of *Aspergillus*. One patient’s bronchoalveolar lavage fluid exhibited a mixture of *Acinetobacter baumannii*, *Candida albicans*, and *Pseudomonas aeruginosa*.

### 3.2. Macroscopic Findings

Upon macroscopic examination, all patients’ lungs displayed an increase in consistency, with patchy patterns observed in 63 cases (79.7%) and pulmonary infarctions observed in 39 cases (49.4%). Upon sectioning and palpation, 62 patients (78.5%) exhibited bloody exudate, 58 patients (73.4%) showed edematous exudate, and 17 patients (21.5%) had purulent exudate. Out of the total cases where lung weight measurements were taken (62 cases), the median combined weight of the right and left lungs was 1748 g, ranging between 610 g to 3005 g. The macroscopic findings of the lungs by age group are summarized in Table 3.

### 3.3. Microscopic Findings

**Figure 2:**
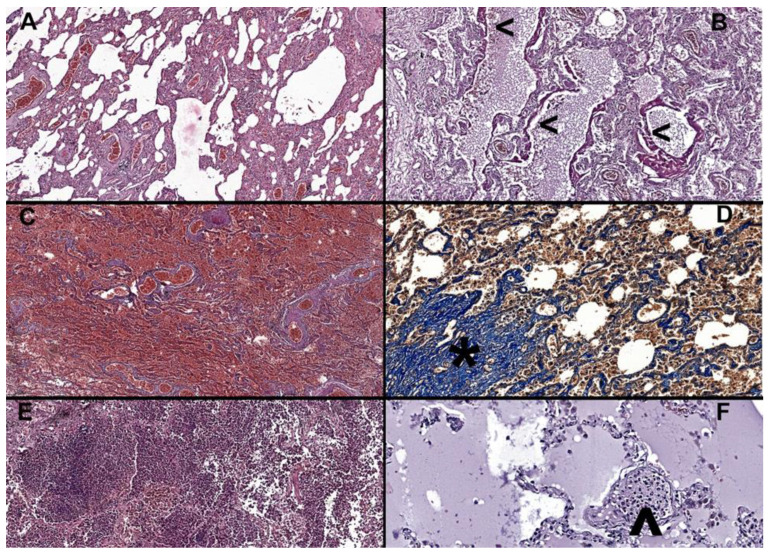
(**A**) Interstitial pneumonia in an 81-year-old male, H&E at 6× magnification, alveolar walls are thickened by inflammatory infiltrate, congestion is marked, and alveolar spaces are deformed. (**B**) Thickened eosinophilic hyaline membranes (**<**) covering the alveolar walls, indicative of organizing phase diffuse alveolar damage (DAD grade 2) in a 69-year-old male, H&E at 10× magnification. (**C**) Widespread pulmonary infarctions with diffuse hemorrhage in a 77-year-old male, H&E at 5× magnification. (**D**) Increased fibrosis with damage to lung structure and formation of fibrous bands and proliferative nodules (*) in a 60-year-old male, trichrome Masson, at 20× magnification. (**E**) Overlapping bronchopneumonia, with abundant neutrophilic infiltrate within alveolar and bronchial space, in an 87-year-old female, H&E at 10× magnification. (**F**) Recently developed microthrombi, comprised of fibrin and inflammatory cells (^) and alveolar edema in an 84-year-old female, H&E at 20× magnification.

Histologically, 42 patients (53.1%) exhibited diffuse alveolar damage (DAD) (Figure 2). Among these patients, twenty-two (27.8%) had DAD in the exudative phase, fifteen (19%) in the organizing phase, and five (6.3%) in the fibrosis phase (Table 4).

In addition, microthrombi (Table 4) were observed in 31 cases (39.2%), with those aged over 70 years, 18 patients (41.9%), being the most affected group. In 29 patients (36.8%), alveolar epithelial desquamation was observed and graded accordingly. Bronchopneumonia (Table 4) was identified in 14 patients (17.7%), while purulent tracheitis was present in twelve (15.2%). Both bronchopneumonia and tracheobronchitis are defined by the presence of neutrophiles in either bronchial or alveolar spaces. Alveolar edema was observed in 66 cases (83.5%), while interstitial pneumonia (Table 4) was present in 59 patients (74.7%). Interstitial pneumonia is defined by the presence of lymphocytes and plasmocytes in the lung interstitium.

Due to inadequate preanalytical processing, the Ashcroft score was evaluated in 76 patients, highlighting interstitial fibrosis in trichrome Masson (Table 4). A median score of 2, ranging from 1 to 3, was observed, with scores ranging from 0 to 6 in all cases, regardless of age group. Alveolar air capacity was quantified, with a median value of 12.5%, ranging from 7% to 20.1%.

### 3.4. Factors Associated with Diffuse Alveolar Damage

Diffuse alveolar damage (DAD) was present in 42 patients and was more frequently observed in patients infected with beta variant (alpha vs. beta: 30 (46.2%) vs. 12 (85.7%), χ^2^ statistics = 7.2, *p*-value = 0.0071). Patients with DAD were significantly younger than those without DAD (with vs. without 67.5 years—IQR [54.3 to 78.5] vs. 77 years [66 to 82], Mann Whitney test: *p*-value = 0.023). No significant association was identified between the presence of DAD and sex (women vs. men: 10 (50%) vs. 32 (54.2%), χ^2^ statistics = 0.1, *p*-value = 0.7428), obesity (obesity vs. non-obesity: 27 (61.4%) vs. 15 (42.9%), χ^2^ statistics = 2.7, *p*-value = 0.1015), or mechanical ventilation (with vs. without mechanical ventilation: 9 (60%) vs. 33 (51.6%), χ^2^ statistics = 0.3, *p*-value = 0.5556). Age and variant proved significant risk factors in both uni- and multivariable regression analysis (Table 5).

## 4. Discussion

Our study showed that the top three lung histopathological biomarkers in patients with fatal COVID-19 are alveolar edema, interstitial pneumonia, and microthrombi, with markers of bronchopneumonia and interstitial edema more frequent in older patients (≥70 years). Overall, a low grade of interstitial fibrosis was observed in our cohort, and diffuse alveolar damage (53.1%) was significantly associated with age and beta variant of the virus.

The most frequently observed comorbidities in our cohort were arterial hypertension and type 2 diabetes mellitus. Although our study found similarities in lung histopathological lesions reported in other studies, the incidence and severity of lung lesions, such as DAD, microthrombosis, and interstitial fibrosis, varied. Our study is the first to use the Ashcroft score to quantify interstitial fibrosis, and it found an overall low grade of interstitial fibrosis, which is different from other studies that did not quantify the phenomenon, especially on a microscopic level.

The percentage of free alveolar space in normal lung tissue varies based on factors such as age, smoking history, and environmental exposures, and it ranges from 70–90% depending on the specific location within the lung and the individual’s age, with the rest of the space occupied by capillaries, elastic fibers, and other structures [24,25]. Our study is the first to use digital slides and image analysis software to microscopically evaluate the percentage of free alveolar space, which was found to be a median of 12.5%.

Our data showed that most patients died in 2020, while only a small percentage died in 2021. The observed shift in the distribution of COVID-19 fatalities from 2020 to 2021 could be attributed to several factors. Changes in treatment options, such as developing new drugs and therapies, may have reduced the number of deaths [26]. Furthermore, increased vaccination efforts could have contributed to decreased COVID-19 deaths [27]. Other factors, such as the patient’s age, underlying comorbidities, and access to medical care, may have also influenced the distribution of deaths between the two years.

Even though most of our participants were men (Table 2), they were statistically significantly younger than women (*p*-value < 0.03). Jin et al. reported a higher COVID-19 mortality rate in men than in women, even though women are, on average, older than men among COVID-19 patients [28]. Yeap et al. reported that men could be more susceptible to severe COVID-19 disease due to higher levels of testosterone and a higher number of comorbidities, such as hypertension, cardiovascular disease, and diabetes [29]. Our results did not support the association between sex and comorbidities (*p*-value > 0.70). Another possible explanation is that women have a stronger immune response to viral infections, including SARS-CoV-2, which could help protect them from the severe form of the disease [30]. Women also have higher levels of estrogen, which has been shown to have anti-inflammatory properties that could potentially reduce the severity of COVID-19 symptoms [31]. Overall, more research is needed to fully understand the reasons for the sex differences in COVID-19 mortality and to develop effective strategies for reducing the impact of the disease on both men and women. In our study, the most observed comorbidities were arterial hypertension, diabetes mellitus type 2, and congestive heart failure. This is consistent with previous research that suggests that individuals with preexisting medical conditions and the elderly are more vulnerable to the life-threatening effects of the virus [32].

The findings in our study revealed that all the lungs showed signs of acute lung injury (Table 3 and Table 4). One of the common findings in SARS-CoV-2 autopsies is an increased consistency of the lungs, present in all cases in our cohort, similarly reported by other autopsy studies [11]. The average lung weight in human autopsies can vary depending on age, sex, height, weight, and smoking history. Grandmaison et al. reported that adults’ average lung weight ranges from 460–800 g per lung, with slight differences based on sex [33]. Increased pulmonary consistency is not a specific or diagnostic feature of COVID-19, as many other lung diseases and conditions can also cause changes in lung density or consistency. In addition, lung weight can vary widely even among healthy individuals, making it difficult to use this measure as a diagnostic tool for lung disease [34]. In our study, the consistency is explained by the lesions associated with ARDS, vascular stasis due to cardiac insufficiency, pulmonary fibrosis, and overlapping bronchopneumonia (Table 4), as described by other histopathological studies of the COVID-19 lung (Table 1) [15,35,36].

In COVID-19, bloody exudate in the lungs can be attributed to a pro coagulative state and microthrombosis [37], causing an inability to drain the blood flow to the heart and systemic circulation, thus leading to a bloody exudate to be found when sectioning the lungs. Ranucci et al. found that COVID-19 patients with ARDS exhibited a procoagulant pattern characterized by elevated levels of D-dimer, fibrinogen, and factor VIII, among other markers suggesting that this procoagulant state may contribute to the development of thrombotic complications in these patients [37]. In our study group, bloody exudate was found more frequently in younger patients (Table 3).

Bronchopneumonia and tracheobronchitis (Table 4) were present in less than 20% of the cases. The two findings can result from bacterial superinfection in patients with SARS-CoV-2 rather than a direct result of the virus-induced lung tissue damage. SARS-CoV-2 primarily targets the respiratory tract and causes lung lesions that predispose to secondary bacterial infections, exacerbating respiratory symptoms and leading to bronchopneumonia and purulent tracheobronchitis. Studies have shown that bacterial superinfection can occur in many patients with COVID-19, particularly those requiring hospitalization or having other underlying health conditions [38,39]. The mechanisms underlying this process are not fully understood but may involve the direct infection of lung cells and the activation of immune responses contributing to tissue damage. Overall, while bacterial superinfection can contribute to the development of bronchopneumonia and purulent tracheobronchitis in patients with SARS-CoV-2, the direct effects of the virus on lung tissue cannot be ruled out, and further research is needed to understand the mechanisms involved.

Interstitial pneumonia is a common finding in severe COVID-19 cases. It is characterized by inflammation and scarring of the tissue between the air sacs in the lungs. Our study identified the presence of interstitial pneumonia (Table 4) in 74.7% of cases, which was similar, albeit higher, to the study conducted by Huang et al., which reported an incidence of 64% [40].

Pulmonary interstitial fibrosis (PIF) is a condition characterized by the scarring of the lung tissue, which can impair lung function; the extent of interstitial fibrosis in COVID-19 patients is still poorly understood and remains an active area of research. Several studies have reported the development of PIF in patients with COVID-19, especially those with severe disease or who require mechanical ventilation [41,42]. In a cohort of 174 COVID-19 patients who underwent chest computed tomography (CT) scans, 25% had evidence of interstitial lung changes, including ground-glass opacities, consolidation, and reticulation, characteristic of PIF [43]. Another study published in European Radiology 2020 reported that PIF was present in 22.4% of COVID-19 patients who required mechanical ventilation [44].

In our research, we have found a degree of interstitial fibrosis (Table 4) in most cases and have used the Ashcroft score [21] to evaluate the extent, as well as counting the number of proliferative fibroblastic nodules present in 25 cm^2^ of lung tissue. The Ashcroft score is commonly used to quantify microscopic interstitial lung fibrosis [21]. Barton et al. examined the post-mortem lung tissue of six patients who died of COVID-19 and reported diffuse alveolar damage and PIF in all cases, with varying degrees of severity [45]. Barton et al. noted that PIF suggests that COVID-19 can cause lung fibrosis, which may contribute to the long-term respiratory complications seen in some COVID-19 survivors [45]. However, the long-term extent of PIF in COVID-19 patients and its impact on lung function and overall health outcomes are still unclear, and further research is needed to understand the relationship between SARS-CoV-2 and PIF and its long-term implications.

We found that half of our patients had DAD (Table 4), lower than reported in a multi-institutional autopsy study conducted in Italy and New York City, where DAD was present in 87% of cases [46]. In our analysis, DAD was more frequently observed in patients infected with the beta variant of the virus than the alpha variant. Moreover, patients with DAD were significantly younger than those without DAD. However, it is important to note that this is a cross-sectional analysis, and the causal relationships between the viral variant, age, and DAD cannot be established based on this study design alone.

The presence of microthrombi (Table 4) was lower in our study, with only 39.2% of patients showing microthrombi compared to 84% in the aforementioned study [46]. Similar findings were reported in other studies from northern Italy, where DAD was present in all cases. Despite the same strain of the virus being present in our region, the reason for this discrepancy is not yet fully understood [14].

In our study, the free alveolar space percentage (Table 4) values were below the normal limits, emphasizing that the lesions induced by COVID-19 severely impact the amount of free alveolar space. Imagistic-based studies suggest the same observations. Fox et al. found that COVID-19 lungs had a marked reduction in the volume of the free alveolar space, which was filled with cellular debris, fibrin, and hyaline membranes [10]. Furthermore, COVID-19 patients had a significant reduction in the volume of the free alveolar space compared to healthy controls, and the degree of reduction in the free alveolar space is positively correlated with disease severity [43]. Different staining methods and image analysis algorithms may yield slightly different results. Therefore, it is essential to interpret histological data in the context of the specific method used to obtain it. Histopathological lesions can provide a more detailed understanding of the severity and extent of SARS-CoV-2 infection.

In our study group, various pathological features, including DAD, microthrombosis, emboli, infarctions, edema, bacterial infection, fibrosis, and interstitial pneumonia, indicate that they likely contribute to the reduction of free alveolar space and compromise respiratory function, leading to death. Therefore, our study implicates these pathological features as potential causes of respiratory insufficiency and subsequent mortality in COVID-19 patients.

Histopathological analysis of autopsy cases can help improve the accuracy of diagnosis, particularly in cases where other diagnostic methods are inconclusive or unavailable, helping clinicians to provide appropriate care and reduce the risk of transmission to others. Autopsy analysis can also provide insights into the long-term effects of SARS-CoV-2 infection, including potential damage to organs and tissues, information that can be used to develop long-term patient monitoring and treatment plans.

Our research stands out in the scientific community due to its unique position as one of the few autopsy-based studies on a substantial number of cases, which supports its originality and scientific value. By conducting autopsies soon after death, we obtained high-quality tissue samples, ensuring an accurate assessment of histopathological biomarkers in fatal cases of coronavirus disease. The scarcity of autopsy studies in this context adds significant value, providing crucial insights into the pathological manifestations and underlying mechanisms of lung involvement. Moreover, including a large case number strengthens the robustness and generalizability of our findings. By employing digital analysis of high-quality tissue samples, we have further enhanced the reliability and validity of our histopathological assessments, leading to more accurate and meaningful conclusions. These factors collectively contribute to the novel histopathological insights that our research provides.

### Limitations of the Study

The study’s retrospective design limited the availability of clinical documentation (e.g., smoking status, alcohol consumption, weight) for patients who died outside hospitals, which may have impacted the accuracy of the data collected. Some tissue fragments could not be evaluated due to inadequate preanalytical processing, including special staining, which may have resulted in incomplete data. The specific variants of SARS-CoV-2 present in the patients included in the study could not be evaluated for each patient because it was not currently determined, which may have limited the conclusions that can be drawn regarding the virus’s impact on the study population. Long-term evaluation of virus-associated histopathological changes could not be evaluated because patients in our cohort did not survive for more than two weeks after the initial COVID-19 infection.

## Figures and Tables

**Table 1:** Lung pathological findings reported in patients with COVID-19.

Authors	Year *	Country	Age Range (Years)	Sample Size	Macroscopic Findings	Microscopic Findings
Fox et al. [10]	2020	US	44–78	10	(a) Pleurisy; (b) patchy pattern edema; (c) pulmonary infarction	(a) DAD; (b) microthrombi
Roden et al. [11]	2020	US	69–94	8	(a) Consolidation; (b) pleurisy; (c) patchy pattern; (d) fibrosis; (e) pulmonary embolism	(a) DAD; (b) squamous metaplasia; (c) bronchopneumonia; (d) pulmonary embolism
Edler et al. [12]	2020	Germany	52–96	80	(a) Congestion; (b) pleurisy; (c) patchy pattern; (d) tracheobronchitis; (e) bronchopneumonia	(a) DAD; (b) squamous metaplasia; (c) bronchopneumonia; (d) fibrosis
Menter et al. [13]	2020	Switzerland	53–96	21	(a) Severe congestion; (b) consolidation; (c) bronchopneumonia	(a) DAD; (b) capillary stasis; (c) bronchopneumonia; (d) interstitial pneumonia; (e) edema; (f) microthrombi; (g) pulmonary embolism
Carsana et al. [14]	2020	Italy	32–86	38	(a) Congestion; (b) edema; (c) patchy pattern	(a) DAD; (b) capillary stasis; (c) bronchopneumonia; (d) interstitial pneumonia; (e) edema; (f) microthrombi; (g) atypical pneumocytes; (h) fibrosis
Lax et al. [15]	2020	Austria	75–91	11	(a) Congestion; (b) emphysema; (c) pulmonary embolism; (d) pulmonary infarction	(a) DAD; (b) bronchopneumonia; (c) interstitial pneumonia; (d) edema; (e) microthrombi; (f) fibrosis;
Suzuki et al. [16]	2022	Japan	28–96	41	(a) Consolidation; (b) tracheobronchitis; (c) pulmonary embolism	(a) DAD; (b) bronchopneumonia; (c) tracheobronchitis; (d) microthrombi
Viksne et al. [17]	2022	Latvia	22–94	88	No data	(a) DAD; (b) microthrombi; (c) pulmonary embolism; (d) fibrosis

* Year of collected data; DAD = diffuse alveolar damage.

**Table 2:** Demographic and comorbidities characteristics of evaluated patients.

Characteristic	All, n = 79 no. (%)	<70 Years, n = 36 no. (%)	≥70 Years, n = 43 no. (%)	Stat. (*p*-Value)
**Sex**				1.2 (0.2721)
Female	20 (25.3)	7 (19.4)	13 (30.2)	
Male	59 (74.7)	29 (80.6)	30 (69.8)	
**Living area**				0.5 (0.4988)
Urban	54 (68.4)	26 (72.2)	28 (65.1)	
Rural	25 (31.6)	10 (27.8)	15 (34.9)	
**Class of BMI**				4.4 (0.2220)
underweight	13 (16.5)	3 (8.3)	10 (23.3)	
normal	22 (27.8)	10 (27.8)	12 (27.9)	
obesity grade 1–2	35 (44.3)	17 (47.2)	18 (41.9)	
obesity grade 3	9 (11.4)	6 (16.7)	3 (7)	
**Comorbidities**				
Arterial hypertension	30 (38)	12 (33.3)	18 (41.9)	0.6 (0.4367)
Type 2 diabetes mellitus	15 (19)	6 (16.7)	9 (20.9)	0.2 (0.6304)
Congestive heart failure	10 (12.7)	4 (11.1)	5 (11.6)	n.a (0.8622)
COPD	7 (8.9)	3 (8.3)	4 (9.3)	n.a (0.8480)
Myocardial infarction	5 (6.3)	0 (0)	5 (11.6)	n.a (0.0381)
Hepatic steatosis	6 (7.6)	2 (5.6)	4 (9.3)	n.a (0.5431)
Malign tumors	7 (8.9)	3 (8.3)	4 (9.3)	n.a (0.8480)
Stroke	6 (7.6)	1 (2.8)	5 (11.6)	n.a (0.1496)

Results are reported as no. (%), where no.= absolute frequency; Stat. is the χ^2^ statistics. Statistically significant *p*-values are highlighted in bold (*p* < 0.05); the remaining values were non-significant. BMI = body mass index; COPD = Chronic obstructive pulmonary disease; n = sample size; n.a. = not applicable, and whenever listed the *p*-value results are from Fisher’s exact test.

**Table 3:** Macroscopic characteristics of lung tissue by age group.

Characteristic	All, n = 79	<70 Years, n = 36	≥70 Years, n = 43	Stat. (*p*-Value)
Combined lung weight, g				1.5 (0.1276) *
median [Q1 to Q3]	1748 [1338 to 2090]	1830 [1505 to 2115]	1645 [1235 to 1865]	
{min to max}	{610 to 3005}	{1225 to 2425}	{610 to 3005}	
Patchy pattern, no. (%)	63 (79.7)	30 (83.3)	33 (76.7)	0.5 (0.468)
Bloody exudate, no. (%)	62 (78.5)	33 (91.7)	29 (67.4)	6.8 (0.0091)
Edema exudate, no. (%)	58 (73.4)	27 (75)	31 (72.1)	0.1 (0.7708)
Purulent exudate, no. (%)	17 (21.5)	5 (13.9)	12 (27.9)	2.3 (0.1311)
Pulmonary infarction, no. (%)	39 (49.4)	20 (55.6)	19 (44.2)	1 (0.3141)

Results are reported as absolute frequency (percentage) for no. (%); * Mann-Whitney test; Stat. is the χ^2^ statistics when the results are reported as no. (%). Q1 = first quartile; Q3 = third quartile; min = minimum; max = maximum.

**Table 4:** Microscopic characteristics of lung tissue by age group.

Characteristic	All, n = 79	<70 Years, n = 36	≥70 Years, n = 43	Stat. (*p*-Value)
**DAD, no. (%)**				n.a. (0.1354)
Absent	37 (46.8)	12 (33.3)	25 (58.1)	
Exudative phase	22 (27.8)	13 (36.1)	9 (20.9)	
Organizing phase	15 (19)	9 (25)	6 (14)	
Fibrosis phase	5 (6.3)	2 (5.6)	3 (7)	
**Lung congestion, no. (%)**				n.a. (0.9935)
Absent	8 (10.1)	4 (11.1)	4 (9.3)	
Slight	22 (27.8)	10 (27.8)	12 (27.9)	
Moderate	29 (36.7)	13 (36.1)	16 (37.2)	
Severe	20 (25.3)	9 (25)	11 (25.6)	
**Microthrombi, no. (%)**	31 (39.2)	13 (36.1)	18 (41.9)	0.3 (0.6022)
**Epithelial desquamation, no. (%)**				n.a. (0.8561)
Absent	50 (63.3)	21 (58.3)	29 (67.4)	
Slight	15 (19)	8 (22.2)	7 (16.3)	
Moderate	10 (12.7)	5 (13.9)	5 (11.6)	
Severe	4 (5.1)	2 (5.6)	2 (4.7)	
**Bronchopneumonia, no. (%)**	14 (17.7)	3 (8.3)	11 (25.6)	4.0 (0.0456)
**Tracheobronchitis, no. (%)**	12 (15.2)	3 (8.3)	9 (20.9)	2.4 (0.1203)
**Alveolar edema, no. (%)**	66 (83.5)	31 (86.1)	35 (81.4)	0.3 (0.5734)
**Interstitial edema, no. (%)**	11 (13.9)	2 (5.6)	9 (20.9)	3.9 (0.0493)
**Antrachotic pigment, no. (%)**				3.6 (0.1688)
Absent	48 (60.8)	25 (69.4)	23 (53.5)	
Slight	16 (20.3)	4 (11.1)	12 (27.9)	
Moderate	15 (19)	7 (19.4)	8 (18.6)	
**Interstitial pneumonia, no. (%)**	59 (74.7)	29 (80.6)	30 (69.8)	1.2 (0.2721)
**Emphysema, no. (%)**	40 (50.6)	17 (47.2)	23 (53.5)	0.3 (0.5790)
**Ashcroft Score**				−0.4 (0.6692)
Median [Q1 to Q3]	2 [1 to 3]	2 [1 to 3]	2 [1 to 3]	
{min to max}	{0 to 6}	{0 to 6}	{0 to 6}	
**Proliferative nodules (no./25 cm^2^)**				−1.2 (0.2348)
Median [Q1 to Q3]	2 [0 to 3.3]	1 [0 to 3]	2 [0 to 4]	
{min to max}	{0 to 11}	{0 to 7}	{0 to 11}	
**Alveolar air capacity**				−0.4 (0.6616)
Median [Q1 to Q3]	12.5 [7 to 20.1]	11.4 [6.8 to 21.5]	12.8 [7.9 to 17.1]	
{min to max}	{1 to 50.5}	{1.8 to 50.5}	{1 to 32.5}	

no. (%) stands for no = absolute frequency; Q1 = first quartile; Q3 = third quartile; min = the lowest value; max = the highest value; Stat. is the χ^2^ statistics when the results are reported as no. (%), Fisher’s exact test when n.a. (not applicable) is displayed, or Z values associated to the Mann-Whitey test otherwise; DAD = diffuse alveolar damage.

**Table 5:** Univariable and multivariable logistic regression model and adjusted OR and associated 95% confidence interval of the presence of DAD.

Variable	Univariable		Multivariable *	
	OR [95%CI]	*p*-Value	OR [95%CI]	*p*-Value
Age, years	0.96 [0.92 to 0.99]	0.0204	0.96 [0.92 to 0.99]	0.0190
Obesity	2.12 [0.86 to 5.23]	0.1036		
Variant, alpha = reference	7.00 [1.45 to 33.80]	0.0154	7.59 [1.51 to 38.10]	0.0138

* Hosmer and Lemeshow Test χ^2^ = 5.77, *p*-value = 0.5663; Nagelkerke R2 = 0.219; model with intercept; Model: log (DAD/(1-DAD) = 3.085–0.046 × ge + 2.027 × Variant (Beta); Percent correct = 62%.

## Data Availability

Data are contained within the article.
